# Effects of Propolis Consumption on Liver Enzymes and Obesity Indices in Adults: A Systematic Review and Dose-Response Meta-Analysis

**DOI:** 10.1016/j.cdnut.2024.104438

**Published:** 2024-08-13

**Authors:** Mohsen Aliakbarian, Mostafa Shahraki Jazinaki, Hossein Bahari, Mohammad Rashidmayvan, Haniyeh Golafrouz, Rozita Khodashahi, Naseh Pahlavani

**Affiliations:** 1 Transplant Research Center, Clinical Research Institute, Mashhad University of Medical Sciences, Mashhad, Iran; 2 Student Research Committee, Mashhad University of Medical Sciences, Mashhad, Iran; 3 Department of Nutrition, Food Sciences and Clinical Biochemistry, School of Medicine, Social Determinants of Health Research Center, Gonabad University of Medical Sciences, Gonabad, Iran; 4 Rajaei Cardiovascular Medical and Research Center, Iran University of Medical Sciences, Tehran, Iran; 5 Clinical Research Development Unit, Imam Reza Hospital, Faculty of Medicine, Mashhad University of Medical Sciences, Mashhad, Iran; 6 Department of Infectious Diseases and Tropical Medicine, Faculty of Medicine, Mashhad University of Medical Sciences, Mashhad, Iran; 7 Health Sciences Research Center, Torbat Heydariyeh University of Medical Sciences, Torbat-e Heydariyeh, Iran; 8 Social Determinants of Health Research Center, Torbat Heydariyeh University of Medical Sciences, Torbat-e Heydariyeh, Iran

**Keywords:** propolis, liver function test, body composition, obesity, meta-analysis

## Abstract

**Background:**

Propolis, a natural resin produced by bees, has been studied for its potential effects on liver enzymes and obesity indices. However, a meta-analysis is necessary to comprehensively understand the impact of propolis on obesity and liver function.

**Objectives:**

This meta-analysis of randomized controlled trials (RCTs) sought to evaluate the effects of propolis consumption on liver enzymes and obesity indices in adults.

**Methods:**

A systematic literature search up to December 2023 was completed in PubMed/Medline, Scopus, and Web of Science, to identify eligible RCTs. Heterogeneity tests of the selected trials were performed using the *I*^2^ statistic. Random-effects models were assessed on the basis of the heterogeneity tests, and pooled data were determined as weighted mean differences (WMDs) with a 95% confidence interval (CI).

**Results:**

A pooled analysis of 24 trials showed that propolis consumption led to a significant reduction in alanine aminotransferase (ALT) (WMD: −2.58; 95% CI: −4.64, −0.52; *P* = 0.01), aspartate aminotransferase (AST) (WMD: −1.84; 95% CI: −3.01, −0.67; *P* = 0.002), and alkaline phosphatase (ALP) (WMD: −24.90; 95% CI: −42.13, −7.67; *P* = 0.005) in comparison with the control group. However, there were no significant effects on gamma-glutamyl transferase (GGT), body weight, BMI (in kg/m^2^), fat mass, body fat percentage, fat-free mass, adiponectin, waist circumference, hip circumference, and waist–hip ratio in comparison with the control group.

**Conclusions:**

We discovered that consuming propolis can lead to a significant decrease in ALT, AST, and ALP levels, without causing significant changes in GGT, anthropometric indices, and adiponectin levels. However, future well-designed RCTs with large numbers of participants and extended durations, focusing on precise propolis dosage and ingredients, are necessary.

## Introduction

Propolis, also known as bee glue, is a lipophilic resin produced by young worker bees (Apis mellifera), which are derived from various plant sources to seal cracks in the hive and thereby protect the colony from infection [1]. Raw propolis typically consists of 50% resins and vegetable balsams, 30% waxes, 10% essential and aromatic oil, 5% pollen, and 5% other bioactive compounds, although this percentage varies depending on the type of propolis and its place of origin [2]. This product is widely used in food, beverages, and nutritional supplements because of its bioactive constituents, including phenolic compounds, flavonoids, terpenes, beta-steroids, resin and aromatic acids [3,4]. A variety of functions, such as anti-inflammatory [5,6], antibacterial [7], antioxidant [8], hepatoprotective [9,10], anticancer [11], and immune activities [12], have been attributed to propolis. Various dosages and forms of propolis supplements are available.

The predicted effects of propolis supplementation on liver biomarkers are inconsistent. No serious side effects or toxicity were reported from the included randomized controlled trials (RCTs). The safety of propolis and its active ingredient has been established through extensive research in both humans and animals [[13], [14], [15]]. According to the study of Zhu et al. [16], elderly people living at high altitudes supplemented with 830 mg/d of Brazilian green propolis experienced a decrease in liver enzyme levels over the course of 2 y. However, Mujica et al. [17] found that providing healthy people with a propolis dose of 13 drops/d failed to decrease their liver enzymes. Also, propolis at 500 mg/d for 4 mo did not significantly affect lipid profiles or glycemic indices in patients with nonalcoholic fatty liver disease (NAFLD) [18]. Caffeic acid phenethyl ester is a component of propolis that has the potential to inhibit the NF-κB signaling pathway and thus produce anti-inflammatory effects [19]. In addition, the effects of propolis supplementation on anthropometric indices remains controversial. Previous interventional studies evaluating the effects of propolis on waist circumference (WC) measurement found no significant effects of propolis on WC [17,[20], [21], [22]]. According to some reports, propolis could help reduce weight gain by regulating transcription factors like sterol regulatory element binding transcription factor 1 (SREBP-1) and SREBP-2, which are involved in fatty acid synthesis and inhibit the accumulation of visceral adipose tissue [23]. However, a previous study among healthy subjects found that 1000 mg raw propolis per day for 60 d significantly increased BMI and weight [24]. A meta-analysis of 5 RCTs conducted recently by Salehi-Sahlabad et al. [25] found that propolis supplementation had no effect on BMI or weight. An earlier meta-analysis that included 14 trials found that taking propolis supplements significantly lowered the levels of both aspartate aminotransferase (AST) and alanine aminotransferase (ALT) but had no effect on anthropometric parameters (such as weight or BMI) [26]. On the other hand, the results of a meta-analysis study that included 6 trials showed that propolis consumption can improve AST levels but not ALT [27].

In recent years, researchers have examined the impact of propolis from different locations on a variety of metabolic parameters in human subjects. However, previous meta-analyses have shown conflicting results from the available limited number of studies until 2019 [[25], [26], [27]]. Also, the previous results were heterogeneous across various outcomes such as liver enzymes, which could have diminished their effectiveness [26]. The duration of supplementation, the region of origin, the amount of propolis used, population characteristics, and the size of the trial sample could all account for the discrepancies in the evidence. Therefore, an updated meta-analysis of studies spanning the years 2017–2023 was performed in order to generate a current estimation of the correlation between propolis and obesity indices and liver enzymes among adults.

## Methods

This systematic review’s steps were based on the proposed PRISMA guideline [28]. The protocol of this meta-analysis is registered in the PROSPERO database under registration ID CRD42023472447**.**

### Search strategy

Medline, Scopus, and Web of Science databases were comprehensively searched until December 2023. This search included no time or language restrictions. The structure of the search strategy consisted of the following mesh and non-mesh terms: (propolis) AND (“intervention” OR “controlled trial” OR “random” OR “randomly” OR “placebo” OR “clinical trial” OR trial OR “randomized controlled trial” OR “randomized clinical trial” OR “rct” OR “blinded” OR “double-blinded” OR “clinical trials” OR trials OR “Cross-Over” OR “parallel”). The reference list of eligible studies was checked to reduce the risk of missing relevant studies, and the Google Scholar search engine was also manually searched.

### Eligibility criteria

Two authors (HB and HG) independently screened the obtained papers using the Endnote 20 software to find eligible studies. The inclusion and exclusion criteria were considered on the basis of the PICOS framework (Population, Intervention, Comparisons, Outcomes, and Study design) [29] (Table 1).

**TABLE 1:** PICOS criteria.

Population	Adults (>18 y old)
Intervention	Propolis intake (without combination therapy)
Comparisons	Placebo intake or nonintervention control
Outcomes	Liver function tests and anthropometric indices
Study design	Randomized controlled trials

The inclusion criteria for this review include *1*) human studies, *2*) interventional studies with RCT design, *3*) intervention with propolis, and *4*) reporting the mean changes and SD in liver function markers and anthropometric indices. Animal studies, studies conducted on the population under 18 y, combination therapy, not reporting the variable changes during the intervention period, not including a control group, observational studies, review articles, and letters to the editor are considered criteria for exclusion from this systematic review.

### Data extraction

Relevant information from the eligible studies, including the name of the first author, the year of publication, the participants’ characteristics (sex, mean age, mean BMI, and health status), the number of individuals, and the type of intervention in each of the groups, characteristics of the propolis intervention (dose and duration time) and the mean changes and standard deviation of the variables during the intervention were extracted independently by 2 researchers (HG and HB). Disagreements were resolved through discussion until consensus was reached.

### Quality assessment

The quality of the included studies was evaluated using the Cochrane Risk of Bias Assessment tool [30]. This tool evaluated the risk of bias across 7 subclasses: random sequence generation, allocation concealment, participant and staff blindness, outcome assessor blinding, incomplete outcome data, selective reporting, and other biases, and the risk of bias in each subclass was then categorized as high, unclear, and low. The general risk of bias was considered as high if there were high risk of bias in ≥2 items or unclear risk of bias in ≥3 items. Disagreements were resolved in consultation with the third author (NP).

### Data synthesis and statistical analysis

The pooled effect size in this meta-analysis was estimated as the weighted mean difference (WMD) and 95% confidence interval (CI) using the random-effects model method proposed by DerSimonian and Laird [31]. If mean changes were not reported during the study, the mean changes were calculated using the following formula: mean change = final values − baseline values. Additionally, SD changes were estimated using the following formula if there was no direct report in the studies: SD = square root [(SD at baseline)^2^ + (SD at the end of study)^2^ − (2r × SD at baseline × SD at the end of study)] [32]. Furthermore, SEs, 95% CIs, and IQRs reported in studies were converted to SDs using Hozo et al. [33]. Heterogeneity among studies was evaluated by Cochran's *Q* test and the I-squared statistic (*I*^2^) [34]. *I*^2^ > 40% or *P* value <0.05 were assumed as significant heterogeneity. Subgroup analysis was performed to identify the source of heterogeneity on the basis of the following predefined criteria [35]: age (>50 and <50), duration of the intervention (<12 and ≥12 wk), propolis dose (˂1000, and ≥1000 mg/d), baseline BMI [normal (18.5–24.9 kg/m^2^), overweight (25–29.9 kg/m^2^), and obese (>30 kg/m^2^)], and health status [healthy, diabetes, NAFLD, polycystic ovary syndrome (PCOS), metabolic syndrome, and others]. The effectiveness of the overall effect size of propolis supplementation on each of the variables from each of the included studies was evaluated by performing a sensitivity analysis using the leave-one-out method [36]. Also, the publication bias of the included evidence was checked for each outcome by executing Egger’s regression, Begg’s rank correlation, and visual interpretation of the funnel plots [37]. Meta-regression was performed to find the source of heterogeneity and investigate the linear relationship between the dose and duration of propolis supplementation with variable changes [38]. Fractional polynomial modeling was used to evaluate the nonlinear relationship between the propolis supplementation features (dose and duration) and outcome changes [39,40]. All analyses were conducted using STATA, version 17 (Stata Corp). *P* values of <0.05 were considered statistically significant for all tests, all of which were 2 tailed.

### Certainty assessment

The certainty of the evidence was assessed using the Grading of Recommendations Assessment, Development, and Evaluation (GRADE) protocol [41]. On the basis of 5 sections of evidence quality, risk of bias [42], inconsistency [42], indirectness [43], imprecision [44], and publication bias [45], were examined. The overall quality of the evidence was graded in 4 levels: low, moderate, high, and very high.

## Results

### Study selection

**FIGURE 1:**
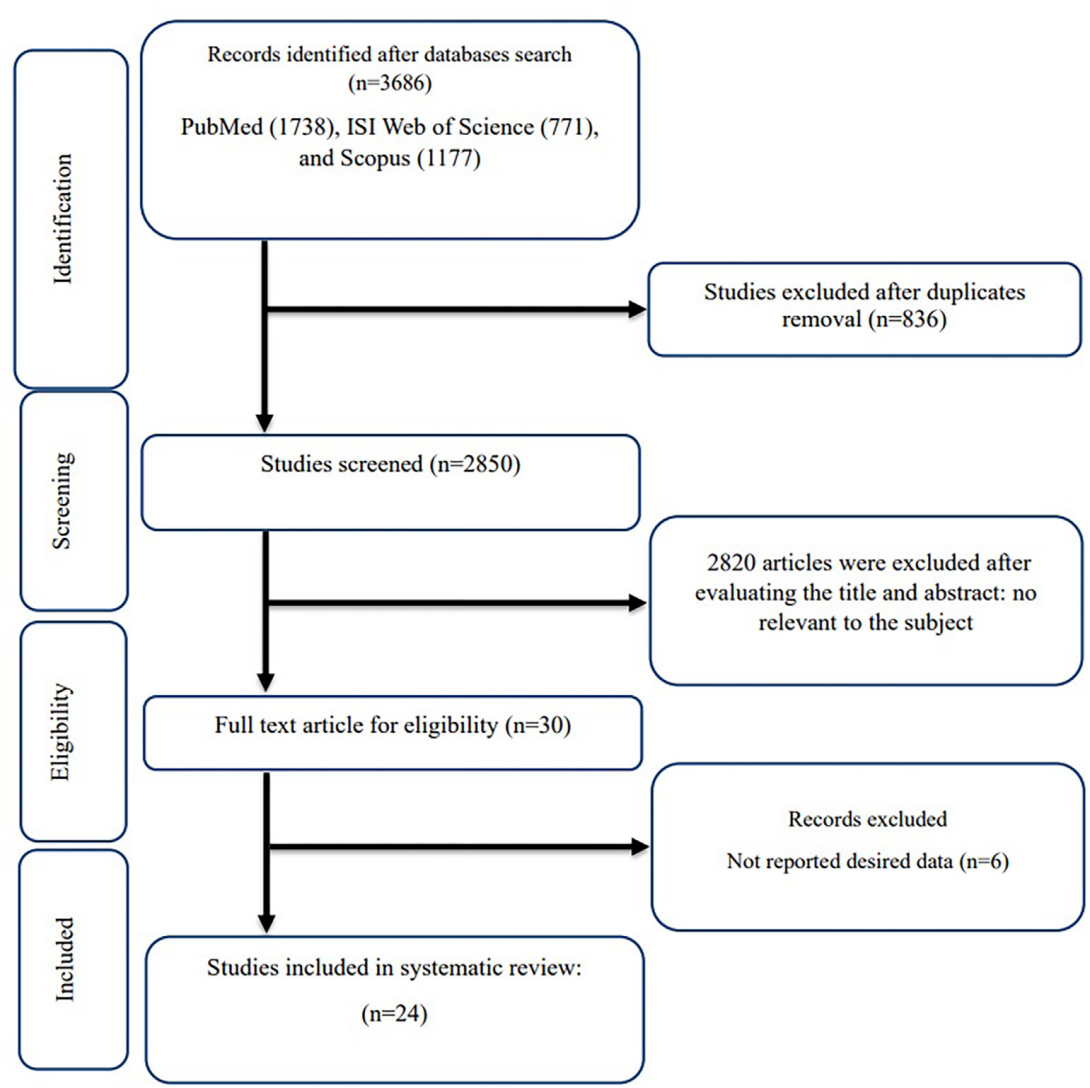
Flowchart of study selection for inclusion trials in the systematic review.

Among the 3686 studies found from the initial search, 836 duplicates were removed. The remaining 2850 studies were screened using their titles and abstracts. Then, the full text of 30 studies was read to evaluate the eligibility criteria, as a result of which 6 studies were excluded because of not reporting the desired data. Finally, 24 studies (25 arms) with a total of 1242 participants were included in this systematic review (Figure 1) [[16], [17], [18],^20^,^22^,[46], [47], [48], [49], [50], [51], [52], [53], [54], [55], [56], [57], [58], [59], [60], [61], [62], [63], [64]].

### Study characteristics

The included studies were published between 2017 [17,46,47] and 2023 [22,[60], [61], [62], [63], [64]]. The studies included in this review were conducted in Chile [17], Iran [18,20,22,46,47,[49], [50], [51],[54], [55], [56],[58], [59], [60], [61], [62]], China [16], Brazil [48], Iraq [52], Indonesia [53], Mexico [57], France [63], and Japan [64]. All included studies had a parallel design except for Sani et al. [63], which had a crossover design. Among the included studies, 3 were conducted on males [50,51,59], 4 on females [58,60,62,64], and the remaining on both sexes. The mean age of the participants ranged from 22 [59] to 72.75 y [16], and their mean BMI was between 23.50 [64] and 33.92 kg/m^2^ [61]. The intervention populations included healthy individuals [16,17,51,52, 59,64] and participants with type 2 diabetes mellitus (T2DM) [20,46,47,49,55,57], chronic kidney disease and proteinuria [48], asthenozoospermia [50], NAFLD [18,54], human immunodeficiency virus [53], irritable bowel syndrome (IBS) [56], breast cancer [58], PCOS [60], obesity and NAFLD [61], T2DM and dyslipidemia [62], insulin resistance and obesity [63], and metabolic syndrome [22]. The type of propolis supplemented in 1 study was in the form of liquid drops [17], and the rest of the studies used tablets and capsules. The dose of propolis supplementation varied from 500 [18,22,48,58,60,62] to 1500 mg/d [20,47,50,54,55,61], and the duration of supplementation was between 4 [51,59] and 48 wk [48]. The characteristics of the included studies are summarized in Table 2.

**TABLE 2:** Characteristic of included studies in meta-analysis.

Studies	Country	Study design	Participant	Sex	Sample size		Trial duration (wk)	Means age		Means BMI		Intervention			Main findings
					IG	CG		IG	CG	IG	CG	Type	Dose (mg/d)	Control group	
Mujica et al. 2017 [17]	Chile	Parallel, R, PC, DB	Healthy individuals	M/F	35	32	12	48	44.5	27.9	28.2	Propolis solution	30 drops	Peppermint + fernet + synthetic	GGT, BW, WC, and BMI did not significantly change in both groups.
Samadi et al. 2017 [46]	Iran	Parallel, R, PC, DB	T2DM	M/F	30	27	12	51.3	56.07	28.18	27.53	Propolis pill	900	Placebo	BW, WC, and BMI did not significantly change in both groups.
Afsharpour et al. 2017 [47]	Iran	Parallel, R, PC, DB	T2DM	M/F	30	30	8	51.81	49.05	26.78	26.74	Propolis capsule	1500	Wheat flour capsule	Propolis reduced the mean AST and ALT levels but was nonsignificant. BW and BMI did not significantly change in both groups.
Zhu et al. 2018 [16]	China	Parallel, R, PC, DB	Elderly living at high altitude	M/F	30	30	24	72.28	73.23	NR	NR	Propolis capsule	830	Placebo	ALT, AST, and GGT did not significantly change in both groups.
Silveira et al. 2019 [48]	Brazil	Parallel, R, PC, DB	CKD + proteinuria	M/F	18	14	48	61.39	61.5	30.58	27.29	Brazilian green propolis tablet	500	Placebo	ALT, AST, and BMI did not significantly change in both groups.
Zakerkish et al. 2019 [49]	Iran	Parallel, R, PC, DB	T2DM	M/F	50	44	12	55.4	54.86	30.04	29.02	Iranian propolis capsule	1000	Placebo	ALP, BW, and BMI did not significantly change in both groups. A notable reduction in ALT and AST in the propolis group was observed.
Hesami et al. 2019 [20]	Iran	Parallel, R, PC, DB	T2DM	M/F	30	30	8	51.81	49.05	26.78	26.74	Propolis capsule	1500	Placebo	BW and BMI did not significantly change in both groups.
Gholaminejad et al. 2019 [50]	Iran	Parallel, R, PC, DB	Asthenozoospermic men	M	29	28	10	31.61	30	27.02	26.52	Propolis capsule	1500	Wheat flour capsule	BW and BMI did not significantly change in both groups.
Soleimani et al. 2021 [18]	Iran	Parallel, R, PC, DB	NAFLD	M/F	27	27	12	42.56	41.85	29.55	28.41	Propolis tablet+ microcrystalline cellulose	500	Placebo	BW and FM were significantly reduced in both groups. The ALP, ALT, AST, and GGT levels in the propolis group were significantly reduced at the end of the trial. FFM did not significantly change in both groups.
Soleimani et al. 2021 [51]	Iran	Parallel, R, PC, TB	Military cadets	M	24	25	4	24.21	24.2	23.82	23.22	Propolis tablet	900	Microcrystalline cellulose	The propolis administration had no effects on BMI, BW, FFM, and FM in subjects within the normal weight range.
Alassaf et al. 2021 [52]	Iraq	Parallel, PC	Healthy subjects	M/F	34	35	8	36.88	39.57	23.67	24.2	Propolis supplement	1000	Placebo	BW and BMI increased significantly in the propolis group.
Triyono et al. 2021 [53]	Indonesia	Parallel, PC, DB	HIV + ARV (antiretroviral treatment)	M/F	19	24	24	36.8	37.1	NR	NR	Propolis capsule	600	Placebo	BW did not significantly change in both groups.
Nikbaf-Shandiz et al. 2022 [54]	Iran	Parallel, R, PC, DB	NAFLD	M/F	23	21	8	38.52	40.14	33.36	33	Propolis capsule+ calorie-restricted diet	1500	Corn starch capsule+ calorie-restricted diet	Between-group differences of ALT, AST, and GGT were not statistically significant at the end of the trial. The BW, BMI, WC, and HC significantly decreased in both groups, whereas the WHR decreased only in the propolis arm.
Afsharpour et al. 2022 [55]	Iran	Parallel, R, PC, DB	T2DM	M/F	30	30	8	51.81	49.05	26.78	26.74	Propolis capsule	1500	Wheat flour capsule	BW and BMI did not significantly change in both groups. Propolis decreased the mean levels of AST and ALT, but it was nonsignificant.
Miryan et al. 2022 [56]	Iran	Parallel, R, PC, DB	IBS	M/F	26	25	6	38.92	44.92	25.61	27.75	Propolis tablet	900	Microcrystalline cellulose tablet	There was no significant change in terms of BW, BMI, and WC in both groups.
Ochoa-Morales et al. 2022 [57]	Mexico	Parallel, R, PC, DB	T2DM	M/F	12	12	12	50	46.7	29	30.2	Propolis capsule	600	Placebo	Propolis administration significantly reduced BW and BMI, but no changes were found in WC.
Davoodi et al. 2022 [58]	Iran	Parallel, R, PC, DB	Breast cancer + chemotherapy	F	26	24	12	49.3	44.36	27.9	27.63	Propolis capsule	500	Starch	BW and BMI did not significantly change in both groups.
Rashvand et al. 2022 [59]	Iran	Parallel, R, PC	Endurance athletes	M	10	12	4	22	22	NR	NR	Propolis capsule	1000	Cellulose	Propolis supplementation had no significant effect on BW of participants.
Abbasi et al. 2023 [60]	Iran	Parallel, R, PC, TB	PCOS	F	28	29	12	18–45	18–45	28.35	26.16	Propolis tablet	500	Placebo	HC was significantly decreased in the propolis group. The BW, BMI, WC, and WHR of the patients in the 2 groups did not significantly change.
Tutunchi et al. 2023 [61]	Iran	Parallel, R, PC, DB	Obesity + NAFLD	M/F	24	24	8	37.5	36.33	34.1	33.75	Propolis capsule + maltodexterine + dietary recommendation	1500	Dietary recommendation	BW, BMI, WC, HC, ALT, and AST levels decreased significantly in both groups.
Moayedi et al. 2023 [62]	Iran	Parallel, R, PC, SB	T2DM + dyslipidemia	F	15	15	8	52.53	53.67	NR	NR	Propolis capsule	500	Placebo	BW and WHR were significantly decreased in the propolis group. Adiponectin was improved after propolis supplementation.
Moayedi et al. 2023 [62])	Iran	Parallel, R, PC, SB	T2DM + dyslipidemia	F	15	15	8	54.07	51.67	NR	NR	Propolis capsule + exercise	500	Exercise	Propolis supplementation significantly improved adiponectin levels and reduced BW and WHR in both groups.
Sani et al. 2023 [63]	France	Crossover, R, PC	Insulin-resistant + obesity	M/F	9	9	12	49	49	31.5	31.7	Propolis	6–9 capsules (250 mg) according to patient's weight	Placebo	No effect on ALT, AST, GGT, BFP, FFM, WC, BMI, and adiponectin levels was reported under propolis supplementation.
Kanazashi et al. 2023 [64]	Japan	Parallel, R, PC, DB	Healthy postmenopausal women	F	25	28	12	75	75	24	23	Propolis capsule	1362	Wheat germ oil capsule	FM was significantly decreased in the propolis group. BFP, FFM, and the level of serum adiponectin were significantly increased in the propolis group.
Sajjadi et al. 2023 [22]	Iran	Parallel, R, PC, DB	Metabolic Syndrome	M/F	33	29	12	54.27	53.86	32.56	34.03	Propolis tablet + microcrystalline cellulose	500	Microcrystalline cellulose	Propolis supplementation could lead to a significant reduction in WC. However, no significant changes were observed in the BW and BMI in both groups.

Abbreviations: ALP, alkaline phosphatase; ALT, alanine transaminase; AST, aspartate aminotransferase; BFP, body fat percentage; BMI; BW, body weight; CG, control group; CKD, chronic kidney disease; CO, controlled; DB, double-blinded; F, female; FFM, fat-free mass; FM, fat mass; GGT, gamma-glutamyl transferase; HC, hip circumference; HIV, human immunodeficiency virus; IBS, irritable bowel syndrome; IG, intervention group; M, male; NAFLD, nonalcoholic fatty liver disease; NR, not reported; PC, placebo-controlled; PCOS, polycystic ovary syndrome; R, randomized; SB, single-blinded; T2DM, type 2 diabetes mellitus; TB, triple-blinded; WC, waist circumference; WHR, waist–hip ratio.

### Quality assessment

Among the included studies, 5 had a high general risk of bias [16,52,53,56,59], whereas the rest had a low general risk of bias. The details of the risk of bias assessment in each subclass are shown in Table 3.

**TABLE 3:** Risk of bias assessment.

Study	Random sequence generation	Allocation concealment	Selective reporting	Other sources of bias	Blinding (participants and personnel)	Blinding (outcome assessment)	Incomplete outcome data	General risk of bias
Mujica et al. 2017 [17]	L	U	L	L	L	U	L	Low
Samadi et al. 2017 [46]	L	U	L	L	L	L	L	Low
Afsharpour et al. 2017 [47]	L	U	L	L	L	L	L	Low
Zhu et al. 2018 [16]	L	U	L	L	U	U	L	High
Silveira et al. 2019 [48]	L	L	L	U	L	U	L	Low
Zakerkish et al. 2019 [49]	L	L	L	L	U	L	L	Low
Hesami et al. 2019 [20]	L	U	L	L	L	U	L	Low
Gholaminejad et al. 2019 [50]	L	L	L	L	L	U	L	Low
Soleimani et al. 2021 [18]	L	L	L	L	L	L	L	Low
Soleimani et al. 2021 [51]	L	L	L	L	L	L	L	Low
Alassaf et al. 2021 [52]	U	U	L	L	U	U	L	High
Triyono et al. 2021 [53]	U	U	H	U	L	U	L	High
Nikbaf-Shandiz et al. 2022 [54]	L	L	L	L	L	U	L	Low
Afsharpour et al. 2022 [55]	L	U	L	L	L	L	L	Low
Miryan et al. 2022 [56]	L	L	H	U	L	U	L	High
Ochoa-Morales et al. 2022 [57]	L	L	L	L	L	U	L	Low
Davoodi et al. 2022 [58]	L	L	L	L	L	U	L	Low
Rashvand et al. 2022 [59]	U	U	H	L	U	U	L	High
Abbasi et al. 2023 [60]	L	U	L	U	L	L	L	Low
Tutunchi et al. 2023 [61]	L	L	L	L	U	U	L	Low
Moayedi et al. 2023 [62]	L	L	L	U	L	U	L	Low
Sani et al. 2023 [63]	L	U	L	L	L	U	L	Low
Kanazashi et al. 2023 [64]	L	L	L	L	L	U	L	Low
Sajjadi et al. 2023 [22]	L	L	L	L	L	U	L	Low

Abbreviations: H, high risk of bias; L; low risk of bias; U, unclear risk of bias.

General risk of bias is considered as high if there were high risk of bias in ≥2 items or unclear risk of bias in ≥3 criteria.

### Meta-analysis

#### The effect of propolis supplementation on ALT levels

**FIGURE 2:**
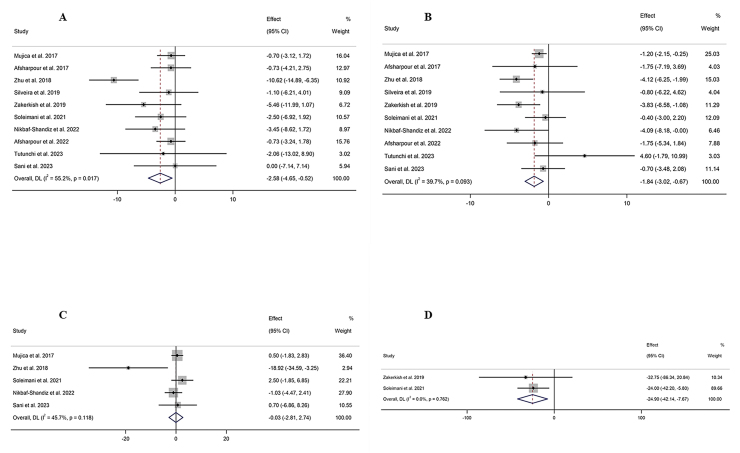

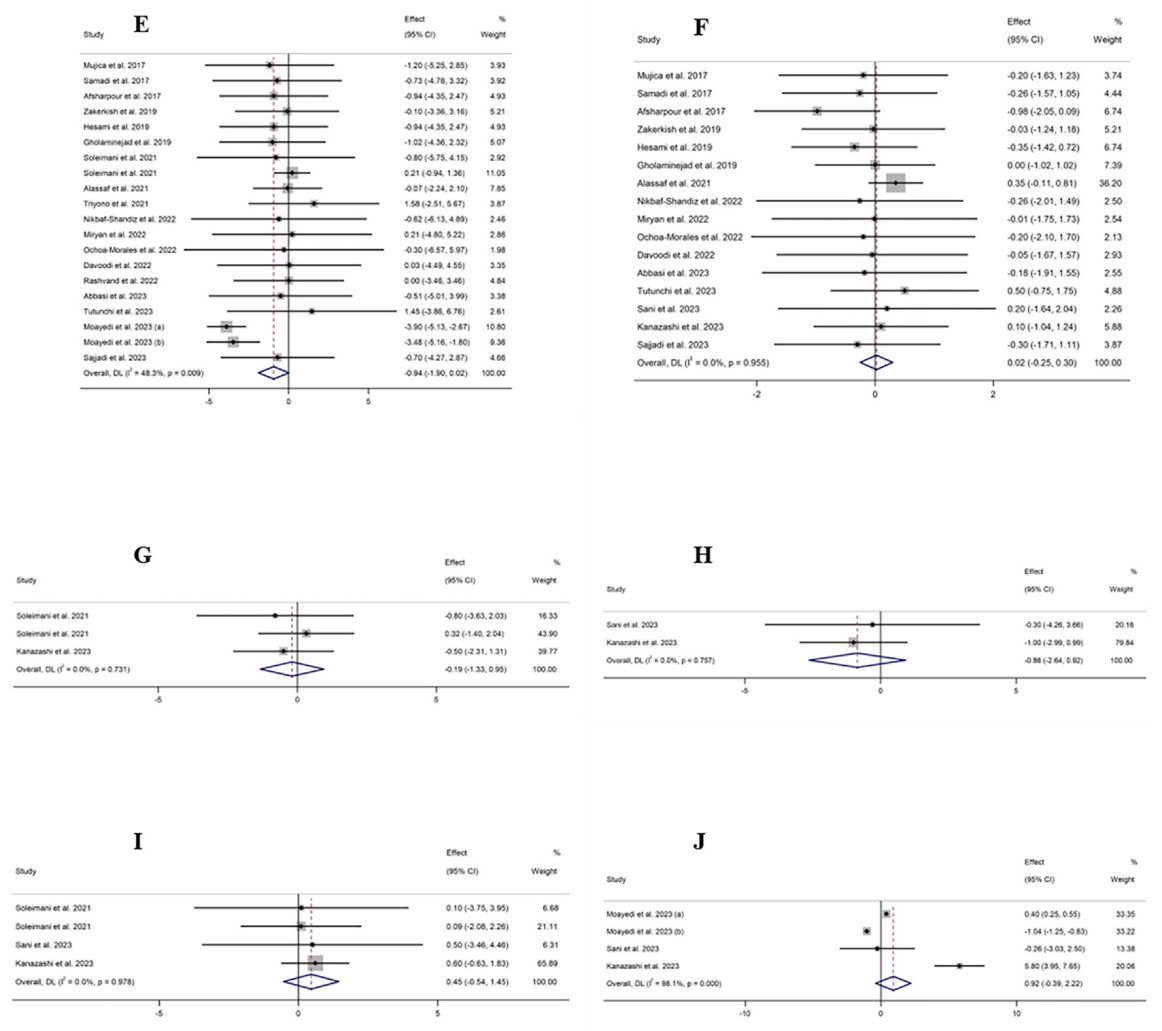

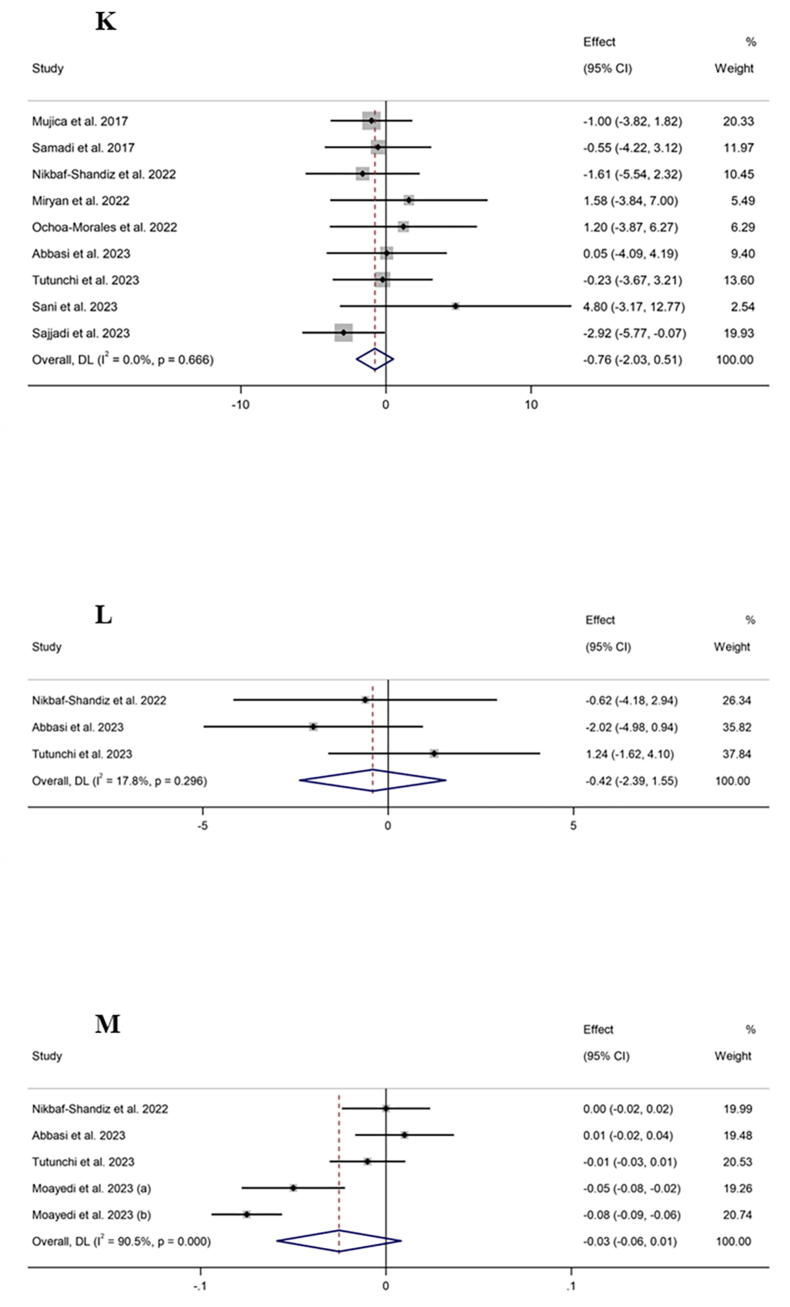
Forest plot detailing weighted mean difference and 95% confidence intervals (CIs) for the effect of propolis intake on (A) ALT (U/L); (B) AST (U/L); (C) GGT (U/L); (D) ALP (U/L); (E) body weight (kg); (F) BMI (kg/m^2^); (G) fat mass (kg); (H) body fat percentage (%); (I) fat-free mass (kg); (J) adiponectin (ug/mL); (K) waist circumference (cm); (L) hip circumference (cm); and (M) waist–hip ratio. ALP, alkaline phosphatase; ALT, alanine aminotransferase; AST, aspartate aminotransferase; BMI; GGT, gamma-glutamyl transferase.

The combination of 10 effect sizes showed that propolis supplementation led to a significant decrease in serum ALT levels compared with control groups (WMD: −2.58 U/L; 95% CI: −4.64, −0.52; *P* = 0.01) (Figure 2A). Also, moderate heterogeneity among the included studies was detected (*I*^2^ = 55.2%; *P* = 0.01). Subgroup analysis, which was performed to find the source of heterogeneity, demonstrated that propolis supplementation did not significantly change serum ALT levels within any predetermined criteria (Table 4).

**TABLE 4:**
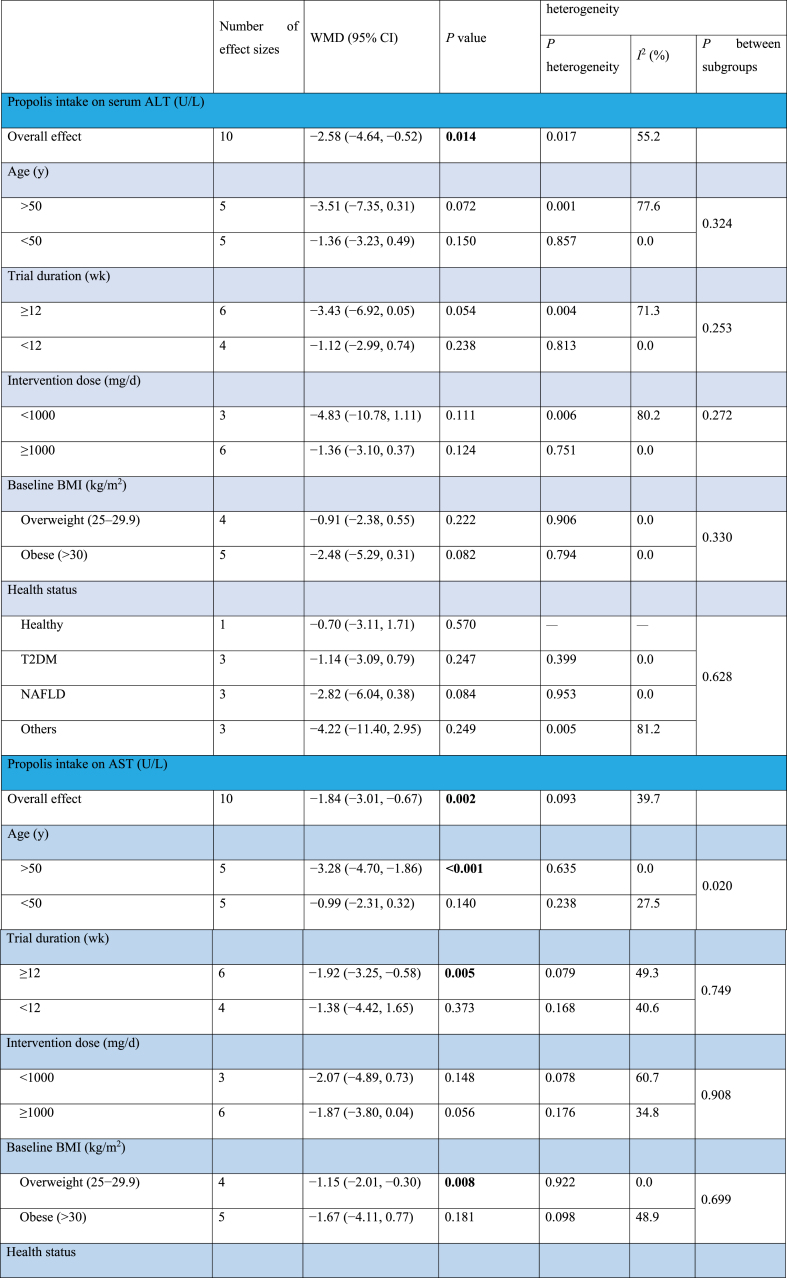

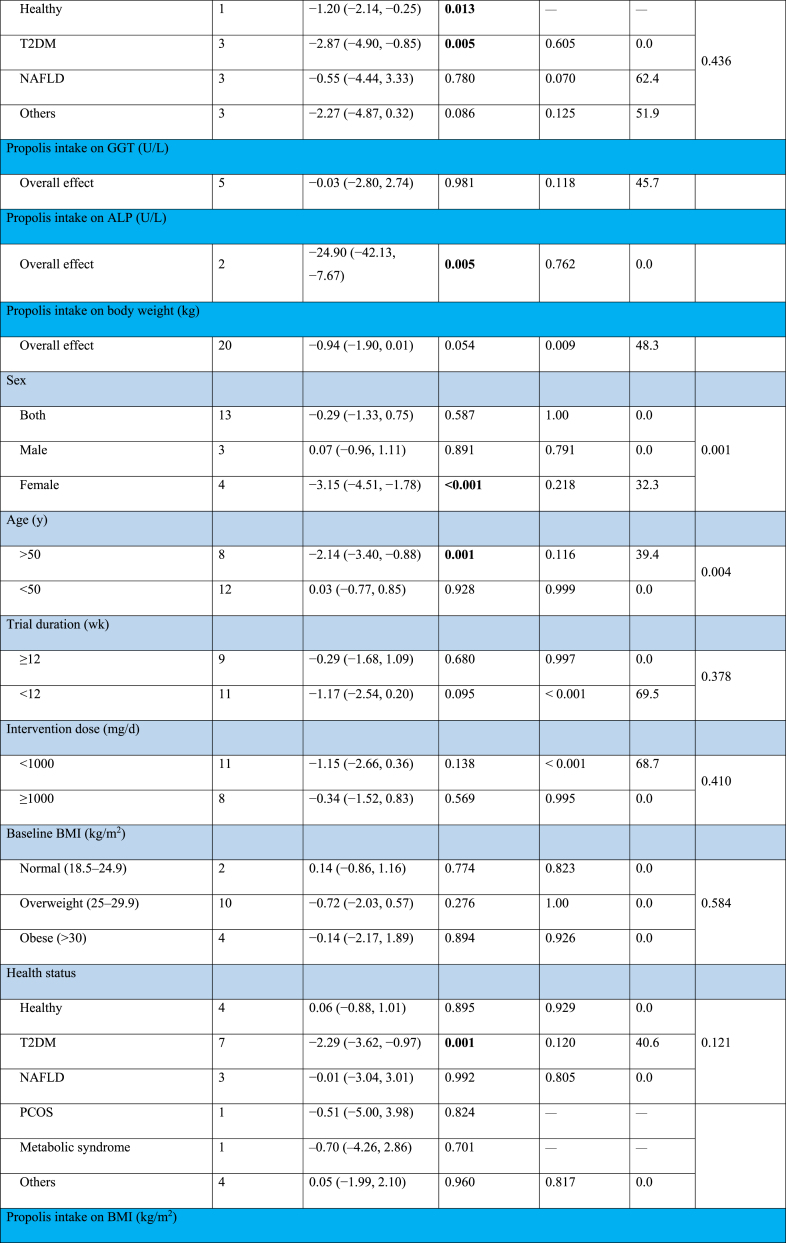

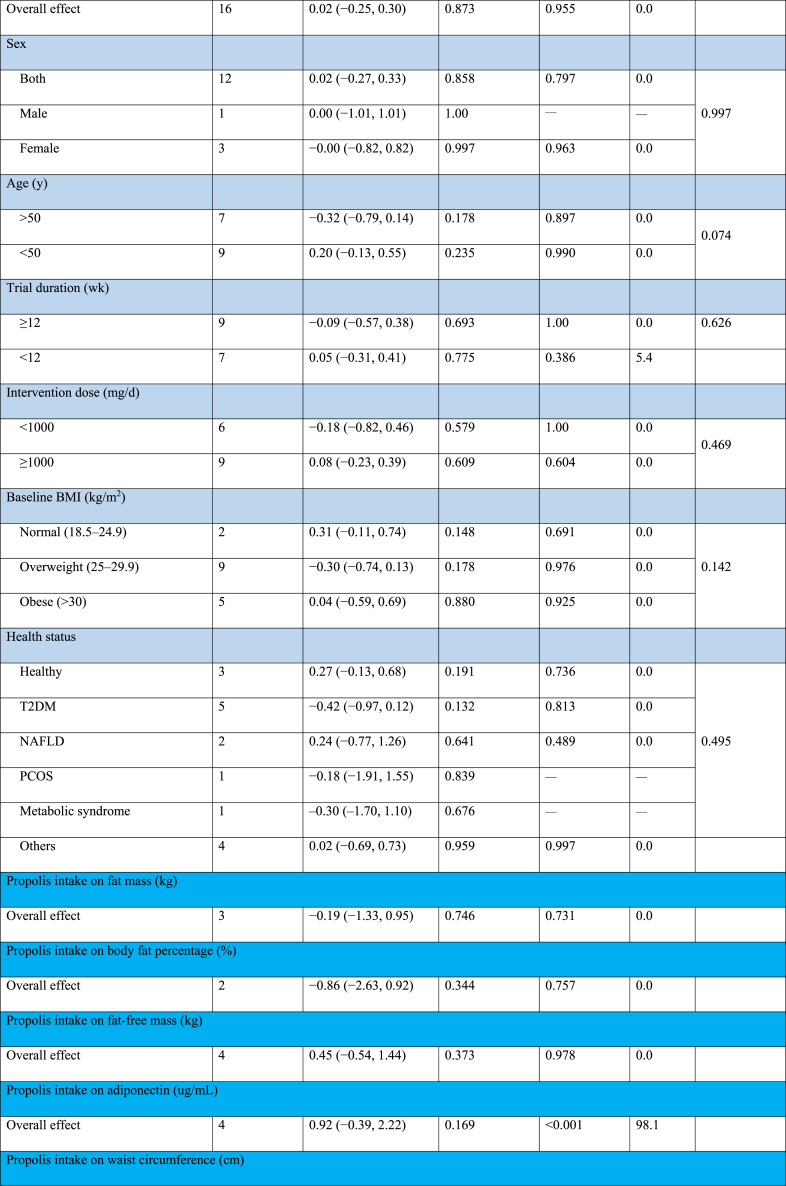

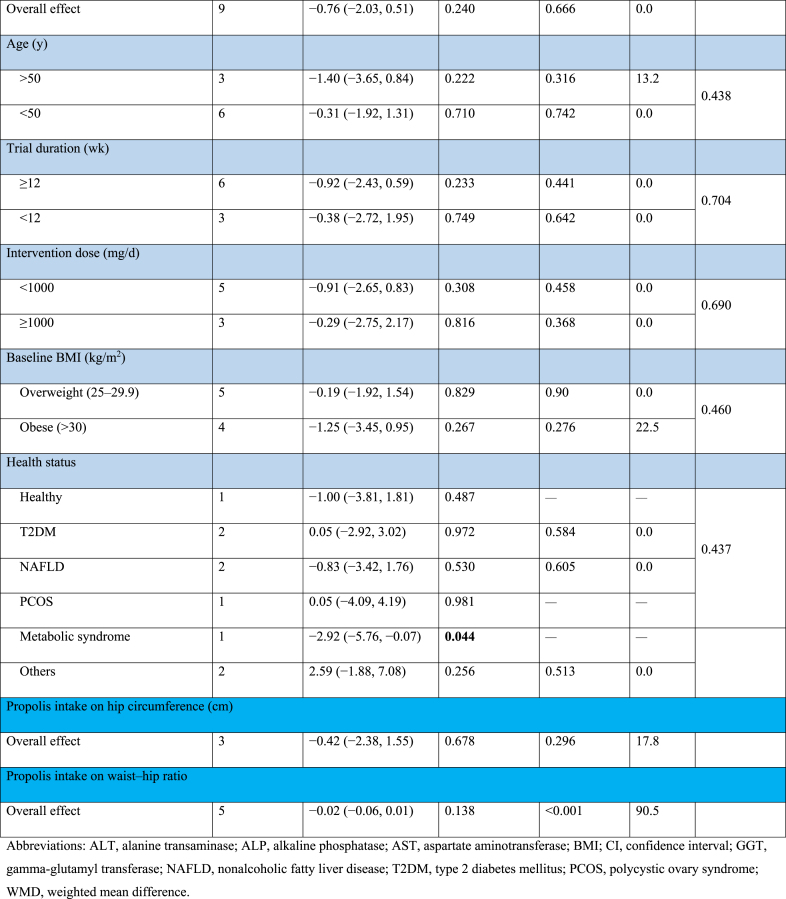
Subgroup analyses of propolis consumption on liver enzymes and anthropometric indices in adults.

#### The effect of propolis supplementation on AST levels

The combination of 10 effect sizes showed that propolis supplementation significantly reduced serum AST levels compared with control groups (WMD: −1.84 U/L; 95% CI: −3.01, −0.67; *P* = 0.002) (Figure 2B). Although there was no significant heterogeneity among the included studies (*I*^2^ = 39.7%; *P* = 0.09), the subgroup analysis mentioned the significant reduction effect of propolis supplementation in overweight, healthy individuals aged >50 y or those with T2DM (Table 4).

#### The effect of propolis supplementation on gamma-glutamyl transferase levels

Meta-analysis of 5 effect sizes demonstrated that propolis supplementation had no significant effect on serum gamma-glutamyl transferase (GGT) levels compared with control groups (WMD: −0.03 U/L; 95% CI: −2.80, 2.74; *P* = 0.98) (Figure 2C). Also, no significant heterogeneity was detected among the included studies (*I*^2^ = 45.7%; *P* = 0.11).

#### The effect of propolis supplementation on alkaline phosphatase levels

The combination of 2 effect sizes showed a significant reduction of serum alkaline phosphatase (ALP) levels following propolis supplementation compared with control groups (WMD: −24.90 U/L; 95% CI: −42.13, −7.67; *P* = 0.005) (Figure 2D). Furthermore, the heterogeneity among the included studies was nonsignificant (*I*^2^ = 0.0%; *P* = 0.76).

#### The effect of propolis supplementation on body weight

The pooling of 20 effect sizes revealed that propolis supplementation had no significant effect on body weight compared with control groups (WMD: −0.94 kg; 95% CI: −1.90, 0.01; *P* = 0.05) (Figure 2E). Although there was significant heterogeneity among the included studies (*I*^2^ = 48.3%; *P* = 0.009). Subgroup analysis demonstrated a significant weight reduction effect for propolis in studies involving female participants aged >50 y with diabetes (Table 4).

#### The effect of propolis supplementation on BMI

After combining 16 effect sizes, it was revealed that propolis supplementation did not lead to a significant change in BMI compared with control groups (WMD: 0.02 kg/m^2^; 95% CI: −0.25, 0.30; *P* = 0.87) (Figure 2F). Also, significant heterogeneity among included studies was not mentioned (*I*^2^ = 0.0%; *P* = 0.95). Subgroup analysis showed that propolis supplementation could not significantly change weight within any of the predefined subgroups (Table 4).

#### The effect of propolis supplementation on fat mass

Performing a meta-analysis on 3 effect sizes emphasized the nonsignificant effect of propolis supplementation on body fat mass compared with control groups (WMD: −0.19 kg; 95% CI: −1.33, 0.95; *P* = 0.74) (Figure 2G). Also, there was no significant heterogeneity among the included trials (*I*^2^ = 0.0%; *P* = 0.73).

#### The effect of propolis supplementation on body fat percentage

The combination of 2 effect sizes showed that propolis supplementation did not significantly change body fat percentage compared with control groups (WMD: −0.86 %; 95% CI: −2.63, 0.92; *P* = 0.34) (Figure 2H). There was no significant heterogeneity among the included studies (*I*^2^ = 0.0%; *P* = 0.75).

#### The effect of propolis supplementation on fat-free mass

Pooling 4 effect sizes revealed that propolis supplementation did not significantly change fat-free mass compared with control groups (WMD: 0.45 kg; 95% CI: −0.54, 1.44; *P* = 0.37) (Figure 2I). No significant heterogeneity was detected among the included studies (*I*^2^ = 0.0%; *P* = 0.97).

#### The effect of propolis supplementation on adiponectin

After combining 4 effect sizes, the nonsignificant effect of propolis supplementation compared with control groups was demonstrated on adiponectin levels (WMD: 0.92 ug/mL; 95% CI: −0.39, 2.22; *P* = 0.16) (Figure 2J), whereas high heterogeneity among included studies was mentioned (*I*^2^ = 98.1%; *P* < 0.001).

#### The effect of propolis supplementation on WC

Meta-analysis on 9 effect sizes showed that propolis supplementation did not lead to a significant change in WC compared with control groups (WMD: −0.76 cm; 95% CI: −2.03, 0.51; *P* = 0.24) (Figure 2K). Furthermore, significant heterogeneity among studies was not included (*I*^2^ = 0.0%; *P* = 0.66). Subgroup analysis revealed a significant reduction effect of propolis on WC in populations with metabolic syndrome (Table 4).

#### The effect of propolis supplementation on hip circumference

The combination of 3 effect sizes showed that propolis supplementation had no significant effect on hip circumference compared with the control groups (WMD: −0.42 cm; 95% CI: −2.38, 1.55; *P* = 0.67) (Figure 2L). In addition, no significant heterogeneity was discovered among the included studies (*I*^2^ = 17.8%; *P* = 0.29).

#### The effect of propolis supplementation on waist–hip ratio

Pooling 5 effect sizes indicated a nonsignificant effect of propolis supplementation compared with the control groups on the WC to hip circumference ratio (WMD: −0.02; 95% CI: −0.06, 0.01; *P* = 0.13) (Figure 2M), whereas there was high heterogeneity among the included studies (*I*^2^ = 90.5%; *P* < 0.001).

### Meta-regression analysis

**FIGURE 3:**
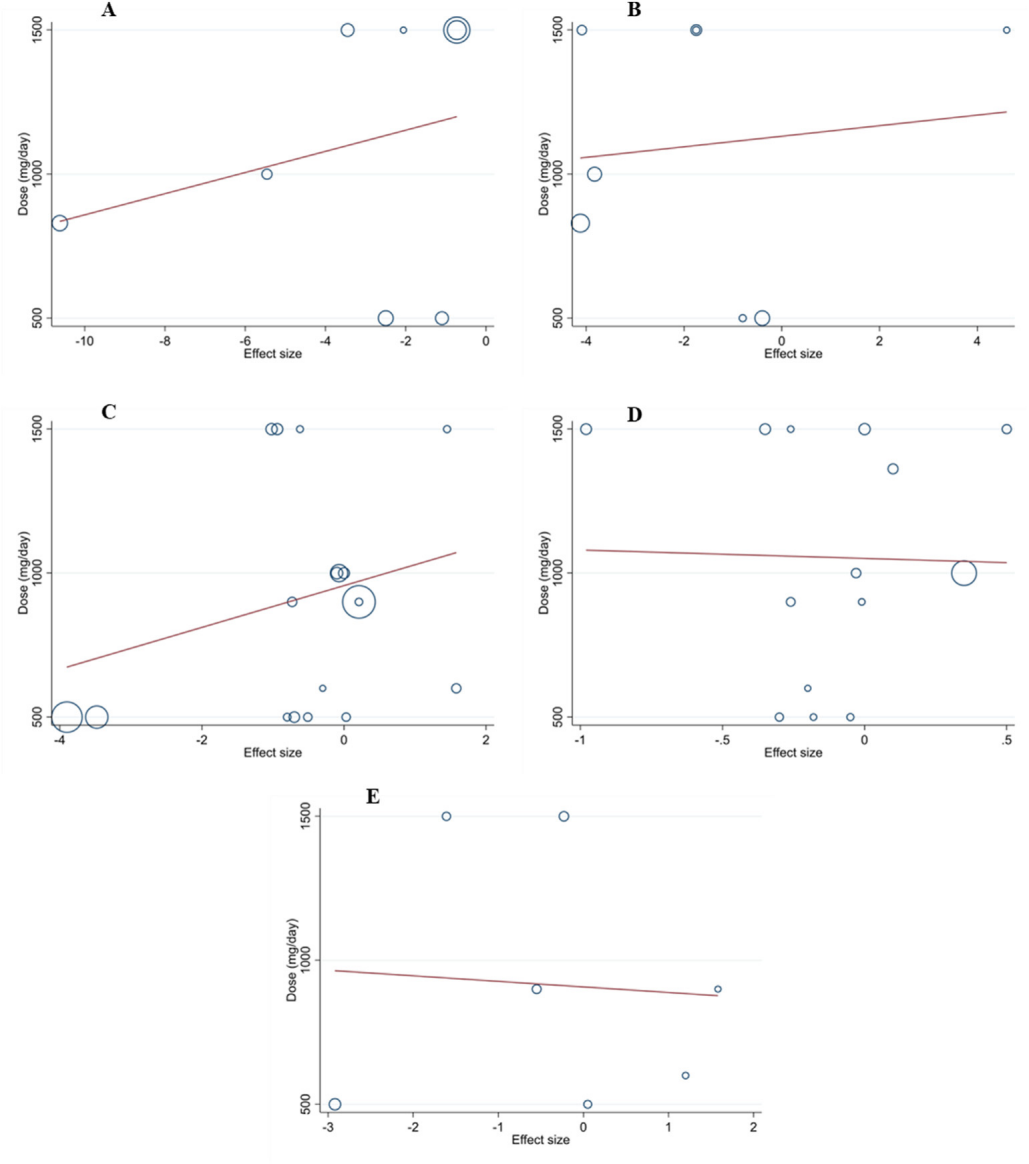
Random-effects meta-regression plots of the association between mean changes in (A) ALT (U/L), (B) AST (U/L), (C) body weight (kg), (D) BMI (kg/m^2^), and (E) waist circumference (cm) and propolis dose. ALT, alanine aminotransferase; AST, aspartate aminotransferase; BMI.

**FIGURE 4:**
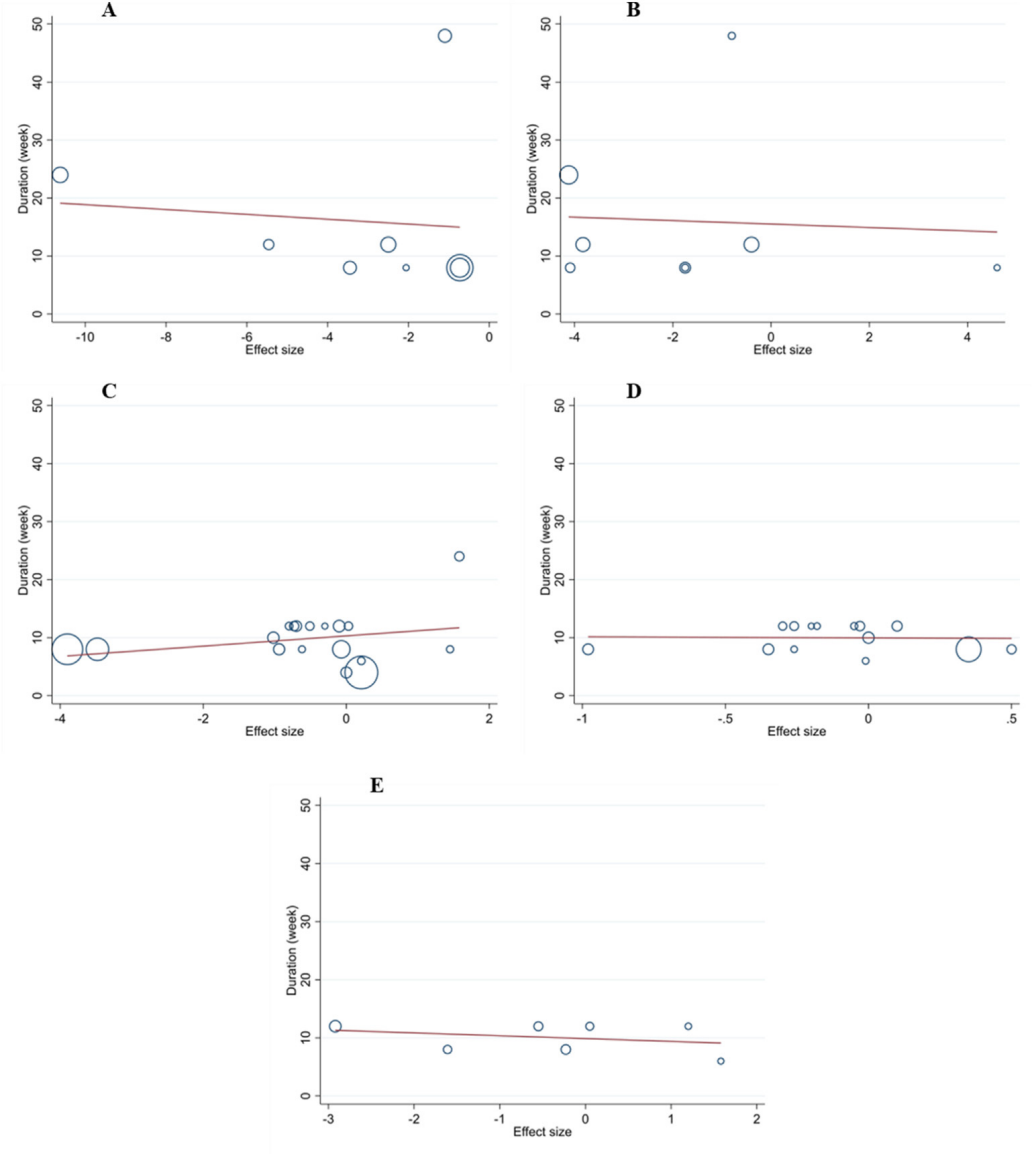
Random-effects meta-regression plots of the association between mean changes in (A) ALT (U/L), (B) AST (U/L), (C) body weight (kg), (D) BMI (kg/m^2^), and (E) waist circumference (cm) and intervention duration. ALT, alanine aminotransferase; AST, aspartate aminotransferase; BMI.

Meta-regression revealed that the dose and duration of propolis supplementation for ALT, AST, body weight, BMI, and WC were not the source of heterogeneity. It also showed no significant linear relationship between the dose and duration of supplementation with changes in these outcomes (FIGURE 3, FIGURE 4).

### Nonlinear dose-response analysis

**FIGURE 5:**
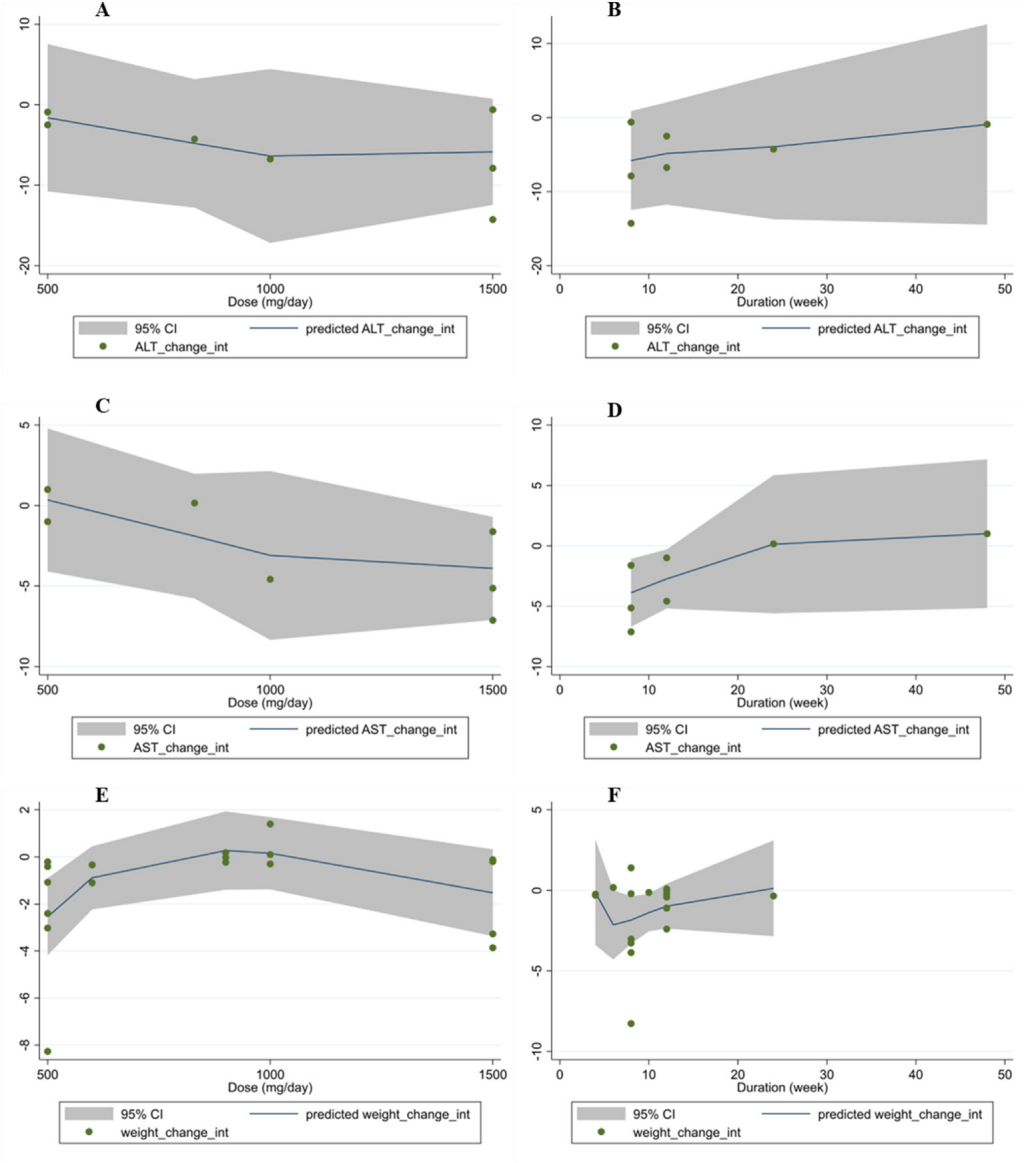

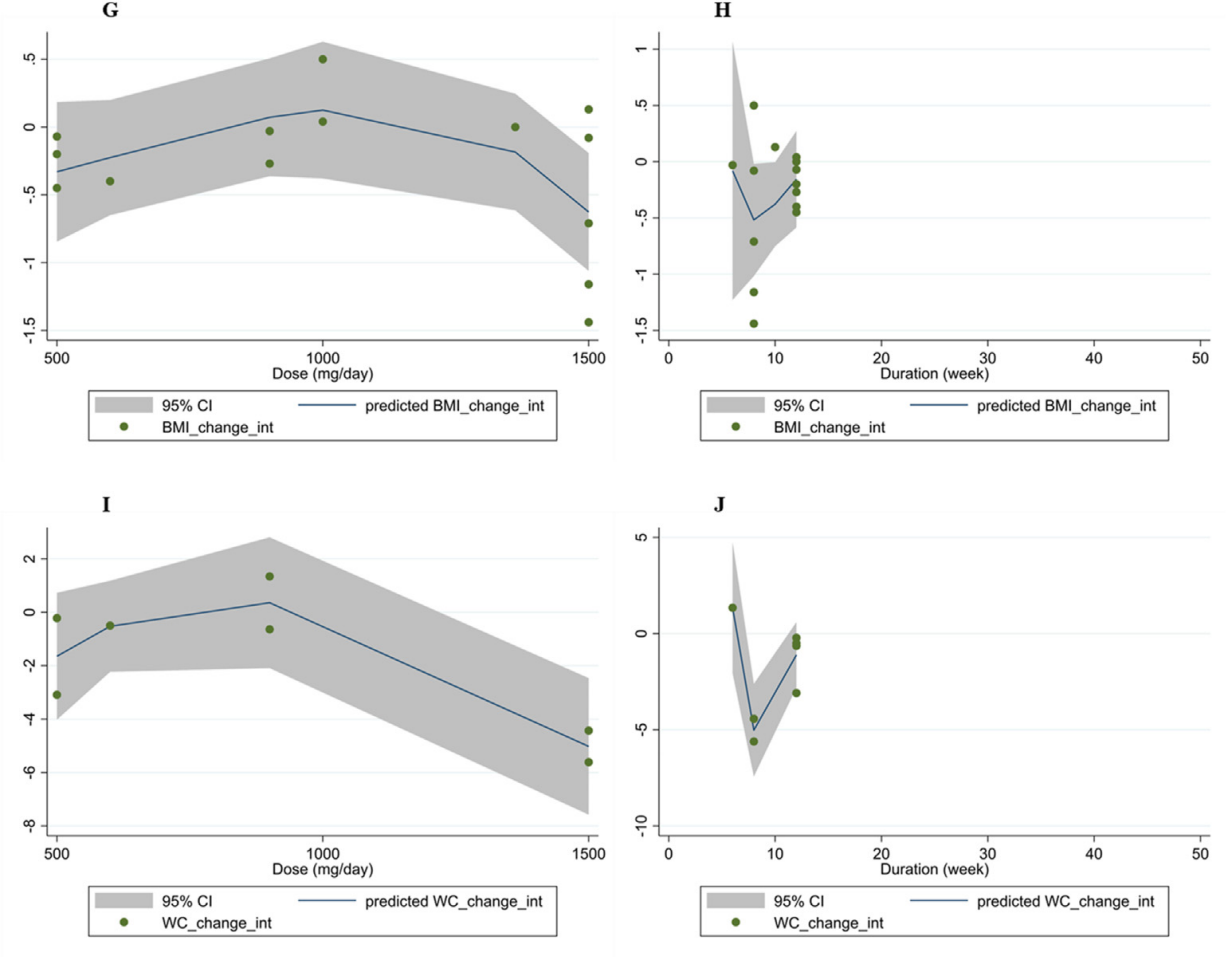
Dose–response relations between propolis dosage (mg/d) and duration (wk) of Propolis supplementation and mean difference in ALT (A, B), AST (C, D), body weight (E, F), BMI (G, H), and waist circumference (I, J). ALT, alanine aminotransferase; AST, aspartate aminotransferase; BMI, body mass index.

Fractional polynomial modeling rejected the existence of a significant nonlinear relationship between propolis supplementation dose and changes in ALT, AST, and BMI. A significant nonlinear relationship was observed between propolis supplement dose (mg/d) and body weight (coefficients = −178.98, *P*_linearity_ = 0.03), and WC (coefficients = −0.002, *P*_linearity_ = 0.02) changes (Figure 5). It seemed that the optimal dose of propolis supplement to reduce body weight and WC was 500 mg/d. Fractional polynomial modeling also identified a significant linear relationship between the duration of supplementation and WC changes (coefficients = −2921.72, *P*_nonlinearity_ = 0.01. The optimal duration of propolis supplementation to reduce WC was 8 wk. However, no significant nonlinear relationship was discovered between the duration of propolis supplementation and changes in other variables.

### Sensitivity analysis

Sensitivity analysis showed that the overall size effect of propolis supplementation on ALT after omitting a study conducted by Zhu et al. [16] (WMD: −1.25 U/L, 95% CI: −2.55, 0.04) for ALP after excluding Soleimani et al. [18] (WMD: −32.75 U/L, 95% CI: −86.33, 20.83) and for body weight after removing Soleimani et al. [51] (WMD: −1.19 kg, 95% CI: −2.13, −0.25), Triyono et al. [53] (WMD: −1.05 kg, 95% CI: −2.02, −0.08), and Tutunchi et al. [61] (WMD: −1.00 kg, 95% CI: −1.97, −0.03) significantly changed. The overall size effect was ineffective for other outcomes of the quality of 1 specific study.

### Publication bias

Begg’s examination and visual analysis of the funnel plots revealed a significant publication bias among the studies examining the effect of propolis supplementation on WC (*p*_Begg_ = 0.009). Although for ALT (*p*_Begg_ = 0.15), AST (*p*_Begg_ = 0.72), GGT (*p*_Begg_ = 0.46), ALP (*p*_Begg_ = 1.00), body weight (*p*_Begg_ = 0.51), BMI (*p*_Begg_ = 1.00), fat mass (*p*_Begg_ = 0.29), body fat percentage (*p*_Begg_ = 1.00), fat-free mass (*p*_Begg_ = 1.00), adiponectin (*p*_Begg_ = 0.73), hip circumference (*p*_Begg_ = 1.00), and waist–hip ratio (WHR) (*p*_Begg_ = 0.46), no evidence of significant publication bias was observed (Supplemental Figure 1).

### GRADE assessment

The evaluation of the certainty of the evidence was done using the GRADE protocol. The grade analysis upgraded the quality of evidence for AST and BMI to very high. Also determined was the certainty of the evidence for ALT, ALP, body weight, fat mass, body fat percentage, fat-free mass, WC, and hip circumference as high quality and for GGT as moderate. However, the quality of evidence investigating the effect of propolis supplementation on adiponectin and WHR was downgraded to low quality. The grade profile is shown in Table 5.

**TABLE 5:** GRADE profile of propolis consumption for liver enzymes and anthropometric indices in adults.

Outcomes	Risk of bias	Inconsistency	Indirectness	Imprecision	Publication Bias	Quality of evidence
ALT	No serious limitations	Serious limitations^1^	No serious limitations	No serious limitations	No serious limitations	⊕⊕⊕◯High
AST	No serious limitations	No serious limitations	No serious limitations	No serious limitations	No serious limitations	⊕⊕⊕⊕ Very high
GGT	No serious limitations	Serious limitations^1^	No serious limitations	Serious limitations^3^	No serious limitations	⊕⊕◯◯ Moderate
ALP	No serious limitations	No serious limitations	No serious limitations	Serious limitations^3^	No serious limitations	⊕⊕⊕◯High
Body weight	No serious limitations	Serious limitations^1^	No serious limitations	No serious limitations	No serious limitations	⊕⊕⊕◯High
BMI	No serious limitations	No serious limitations	No serious limitations	No serious limitations	No serious limitations	⊕⊕⊕⊕ Very high
Fat mass	No serious limitations	No serious limitations	No serious limitations	Serious limitations^3^	No serious limitations	⊕⊕⊕◯High
Body fat percentage	No serious limitations	No serious limitations	No serious limitations	Serious limitations^3^	No serious limitations	⊕⊕⊕◯High
Fat-free mass	No serious limitations	No serious limitations	No serious limitations	Serious limitations^3^	No serious limitations	⊕⊕⊕◯High
Adiponectin	No serious limitations	Very serious limitations^2^	No serious limitations	Serious limitations^3^	No serious limitations	⊕◯◯◯ Low
Waist circumference	No serious limitations	No serious limitations	No serious limitations	No serious limitations	Serious limitations^4^	⊕⊕⊕◯High
Hip circumference	No serious limitations	No serious limitations	No serious limitations	Serious limitations^3^	No serious limitations	⊕⊕⊕◯High
Waist–hip ratio	No serious limitations	Very serious limitations^2^	No serious limitations	Serious limitations^3^	No serious limitations	⊕◯◯◯ Low

1 There is high heterogeneity (*I*^2^ > 40%).

2 There is high heterogeneity (*I*^2^ > 75%).

3 The sample size is <400.

4 There is a significant publication bias based on Egger’s test.

## Discussion

The present study examined the effects of propolis on liver enzymes and obesity-related indices, and a total of 24 studies were included, on the basis of which propolis was able to decrease ALT and AST significantly, but its effects on GGT and ALP and obesity-related indices including weight, BMI, fat mass, body fat percentage, fat-free mass, WC, hip circumference, WHR, adiponectin were not statistically significant.

It has been shown that the increased level of liver enzymes is associated with inflammation and accumulation of fat in the liver and may lead to NAFLD; therefore, 1 of the approaches to prevent this from happening is to use supplements that are based on natural compounds [[65], [66], [67], [68]]. On the basis of the results of our study, we found that propolis caused a significant decrease in ALT and AST levels, but its effects on GGT and ALP even though there was a decrease were not statistically significant. The subgroup analysis showed a significant decrease in AST levels in subjects over 50 y old, overweight individuals, healthy, and T2DM patients. The hepato-protective effects of propolis have been shown in various previous cell and animal studies [[69], [70], [71]]. In line with our findings, Hallajzadeh et al. [26] showed in a systematic review and meta-analysis that propolis can significantly reduce ALT and AST levels. Also, similar results were obtained in a clinical trial study and propolis supplementation for 2 y with a dose of 830 mg/d decreased liver enzymes (a significant decrease in ALT, a decreasing but nonsignificant trend in AST and GGT levels) in the elderly subjects [16]. In another systematic review and meta-analysis study that included 6 articles, it was shown that propolis consumption significantly reduces AST, but its effects on ALT were nonsignificant [27]. In Zakerkish et al. [49] study, administration of Iranian propolis (1000 mg/d during 90 d) in patients with T2DM could significantly reduce ALT and AST levels, which confirms the findings of our study. In a clinical trial study that was conducted on obese subjects with NAFLD, propolis supplementation in combination with diet modification at a dose of 1500 mg/d for 8 wk caused a marginal decrease in the level of liver enzymes compared with other study groups [61]. It seems that because of the fact that in some chronic diseases such as type 2 diabetes and NAFLD, liver enzymes undergo changes and their levels increase [72]; therefore, propolis supplementation in disease conditions can have a greater effect on the level of these enzymes. Therefore, this case should be considered in future studies. Contrary to these results, in 1 study conducted by Silveira et al. [48], Brazilian green propolis extract in patients with chronic kidney disease at a dose of 500 mg/d for 12 mo did not have a significant effect on liver enzymes [48], which is similar to these results in the study of Sani et al. [63], where it was shown that poplar propolis extract powder in 250 mg capsules contains 70% propolis concentrate, 15% magnesium stearate, 10% silicium dioxide, and 5% carob powder, in obese non-diabetic insulin-resistant individuals after 3 mo could not significantly improve ALT and AST levels. Probably, the reason for the difference in the results of the various studies can be the dose of propolis given, the place from which the propolis was extracted, the extraction method, different forms of propolis supplementation (solution/pill/capsule), duration, age, and the health status of the participants.

Considering the fact that increased fat accumulation in the liver can cause hepatic-inflammation and fatty liver, and on the other hand, the increased level of lipid profile can be related to fatty liver, it seems that 1 of the mechanisms of the effect of propolis in improving hepatic enzymes is reduced tissues fat accumulation [73,74]. Probably, the flavonoids in propolis reduce cholesterol synthesis by inhibiting hepatic acyl CoA cholesterol o-acyltransferase and 3-hydroxy-3-methylglutaryl-CoA reductase [75]. Another possible mechanism of the protective effect of propolis on the liver is related to SREBP-1 responsive lipogenic genes, Stearoyl-Coenzyme A desaturase 1, and Fatty acid-binding protein 5, which increase fat oxidation and reduce its accumulation in the liver [[76], [77], [78]]. Also, because of having a wide range of antioxidants and flavonoids such as galangin, naringin, pinocembrin, and chrysin, propolis can be effective in improving liver enzymes [77].

The results of the present study showed that consumption of propolis does not have a significant effect on obesity-related factors such as weight, BMI, fat mass, body fat percentage, fat-free mass, WC, hip circumference, WHR, and adiponectin. Similar to the present study, in Salehi-Sahlabadi et al. [25] meta-analysis study that was conducted on 5 articles, receiving propolis had no significant effect on body weight and BMI; the results of this study confirm our findings despite the small number of included studies. In line with our findings, Miryan et al. [56] study revealed that propolis supplementation in patients with IBS (900 mg/d after 6 wk) had no significant effects on weight, BMI, and WC [56]. Also, in a study conducted by Soleimani et al. [18], propolis supplementation for 16 wk (900 mg/d) in NAFLD patients had no significant effect on weight, fat-free mass, and body fat mass. Also, in a study conducted on patients with T2DM, Afsharpour et al. [79] showed that propolis intake for 8 wk at a dose of 1500 mg/d had no significant effect on weight and BMI, which was similar to the results of Mujica et al. [17]. However, contrary to the results of most of the studies conducted on the effects of propolis on anthropometric indices, in 1 study, Samadi et al. [46] showed that consumption of propolis at a dose of 900 mg/d for 12 wk caused a significant reduction in weight and BMI in patients with T2DM; perhaps, the reason for this inconsistent result is the lack of adjustment of confounding factors such as physical activity level, medicine, and food intake in this study. It seems that considering that anthropometric indices change later than serum factors, in order for propolis to significantly change them, it should be received with a high dose and for a long time along with dietary modifications and increased physical activity.

In Sajjadi et al. [22] study, propolis supplementation extract with a dose of 500 mg/d for 12 wk in patients with metabolic syndrome caused a significant reduction in WC. In an interventional study, consumption of propolis (500 mg/d) in combination with training in women patients with T2DM increased the adiponectin levels after 8 wk of intervention [62]. However, in Rashvand et al. [59] study, consumption of propolis at a dose of 500 mg/d for 4 wk in male athletes had no significant effect on body weight. The reason for this difference in the results of the studies despite giving similar doses is probably because of the different designs of the studies as well as the difference in the effective ingredients of the propolis supplement. It is quite clear that propolis can have beneficial effects on health because of having >300 effective compounds including flavonoids, caffeic acid phenethyl ester, polyphenols, amino acids, and vitamins, which mainly have multiple antibacterial, antioxidant, and anti-inflammatory roles [80]. It seems that propolis can be used as an adjunctive therapy along with other interventions such as diet modification and exercise to improve obesity-related indices for a long duration.

Considering that propolis as a health-promoting supplement and an adjunctive treatment can be useful in improving some factors related to chronic diseases, it is recommended that in future studies the exact doses of propolis by specifying its effective ingredients should be tested individually in health and disease conditions to determine its exact functions of this natural compound.

This current meta-analysis is the first study to have comprehensively examined the propolis intake effects on all anthropometric factors, body composition indices and liver function tests with a high-quality methodological approach and a large number of included studies. Also, in the studies that were included, only a few of them had a high general risk of bias. However, the present work had some limitations: First, studies that were included in the analysis were conducted on subjects with various health and disease conditions. Second, studies have been conducted on various types of propolis from different regions of the world, and the method of extracting them was varied, which can affect propolis composition and the results of the studies. Future well-designed long-term studies with large sample sizes and special propolis doses are required to evaluate the precise impacts of propolis on anthropometric and body composition indices.

In conclusion, in the current meta-analysis, we found that propolis supplementation significantly decreases the hepatic enzyme levels of ALT and AST in adults. Also, a significant decreasing effect of propolis intake on GGT and ALP was not found in our analysis. As well as propolis consumption was not associated with significant changes in the levels of anthropometric indices and adiponectin levels. However, because of the lack of side effects, propolis can be taken in doses between 500 and 1000 mg/d as a health-promoting supplement alongside diet modification. Furthermore, well-designed RCTs, particularly those with a low risk of bias, are needed to assess the precise effects of propolis supplementation on anthropometric and body composition indices.

## Acknowledgments

We would like to thank the Transplant Research Center, Mashhad University of Medical Sciences, for providing support in this manuscript.

## Author contributions

The authors’ responsibilities were as follows – RK, HB: conceived and designed the research; MSJ, HG: performed screening and data extraction; MSJ, HB: analyzed data; MR, NP, MSJ: drafted the manuscript; NP, MA revised the manuscript; and all authors: read and approved the final manuscript.

## Conflict of interest

The authors report no conflicts of interest.

## Funding

The authors reported no funding received for this study.

### Data availability

All data generated or analyzed during this study are included in this published article.
