# Supplementary material for: Effects of Propolis Consumption on Liver Enzymes and Obesity Indices in Adults: A Systematic Review and Dose-Response Meta-Analysis
Source: Curr Dev Nutr. 2024 Aug 13;8(9):104438. doi: 10.1016/j.cdnut.2024.104438 (PMC11407979; doi:10.1016/j.cdnut.2024.104438)
Supplement: Multimedia component 1 [file mmc1.docx]

**Effects of Propolis Consumption on** **Liver Enzymes and Obesity Indices in Adults: A Systematic Review and dose-response Meta-analysis**

**Mohsen Aliakbarian**

Transplant Research Center, Clinical Research Institute, Mashhad University of Medical Sciences, Mashhad, Iran.

A

B

C

D

E

F

G

H

I

J

K

L

M

**Supplementary Figure 1.** Funnel plots for the effect of Propolis intake on A) ALT (U/L); B) AST (U/L); C) GGT (U/L); D) ALP (U/L); E) body weight (kg); F) BMI (kg/m²); G) fat mass (kg); H) body fat percentage (%); I) fat-free mass (kg); J) adiponectin (ug/ml); K) waist circumference (cm); L) hip circumference (cm); and M) waist-hip ratio.
